# Improved Progression-Free Survival Associated with Tumor-Infiltrating Lymphocytes in High-Grade Endometrial Cancer

**DOI:** 10.3390/jcm12020603

**Published:** 2023-01-11

**Authors:** Chun-Ting Fan, Shih-Tien Hsu, Lou Sun, Sheau-Feng Hwang, Chih-Ku Liu, Yu-Hsiang Shih, Ming-Jer Chen, Hsin-Ni Li, Jun-Sing Wang, Mei-Chin Wen, Chien-Hsing Lu

**Affiliations:** 1 Department of Obstetrics and Gynecology, Taichung Veterans General Hospital, Taichung 40705, Taiwan; 2 School of Medicine, National Yang Ming Chiao Tung University, Taipei 11221, Taiwan; 3 Department of Pathology and Laboratory Medicine, Taichung Veterans General Hospital, Taichung 40705, Taiwan; 4 Division of Endocrinology and Metabolism, Department of Internal Medicine, Taichung Veterans General Hospital, Taichung 40705, Taiwan; 5 Department of Post-Baccalaureate Medicine, College of Medicine, National Chung Hsing University, Taichung 40227, Taiwan; 6 Institute of Biomedical Sciences and Rong-Hsing Research Center for Translational Medicine, National Chung-Hsing University, Taichung 40227, Taiwan; 7 Division of Pathology, China Medical University Hsinchu Hospital, Hsinchu 30272, Taiwan

**Keywords:** endometrial cancer, tumor-infiltrating lymphocyte, mismatch repair, survival outcome

## Abstract

Tumor-infiltrating lymphocytes (TILs) have emerged as a prognostic marker in endometrial cancer (EC). However, the role of TILs in EC with distinct histology grades and molecular types (such as mismatch repair [MMR] deficiency) has not yet been made clear. We retrospectively included 237 patients with primary EC who underwent a standard staging operation of laparoscopic or laparotomy total hysterectomy and bilateral salpingo-oophorectomy for analyses. An independent pathologist who was blind to the study patients’ information reviewed the pathologic slides to assess TILs according to the method introduced by the International Immuno-Oncology Biomarkers Working Group in 2017. The outcomes of interest included both progression-free survival (PFS) and overall survival (OS). The Kaplan–Meier method was used to determine the curves of PFS and OS according to TILs, and also in the relevant subgroups (low-grade vs. high-grade, MMR-proficient vs. MMR-deficient). After a median follow-up duration of 1.82 years, 18 patients had experienced either disease progression or death. Overall, TILs (+) were not associated with PFS or OS. We did observe, however, that TILs (+) were associated with a better PFS (*p* = 0.045) in patients with high-grade EC, but not in those with low-grade tumors (*p* = 0.733). The effect of TILs on PFS was not observed in patients with MMR-proficient (*p* = 0.347) or MMR-deficient (*p* = 0.168) EC. TILs were associated with a better PFS in patients with high-grade EC. Our results suggest that TILs may be a potential prognostic marker in these patients.

## 1. Introduction

Endometrial cancer (EC) is one of the most common gynecologic cancers in developed countries. Its incidence has been increasing globally, with 417,000 new cases and 97,000 deaths in 2020 [1,2]. Risk factors of EC included obesity, early menarche, late menopause, nulliparity, and age [3,4]. The increase in the incidence of EC [1,2] may be partly attributed to the rising trend of obesity [5] and the increase in the aging population [6]. Encouraging physical activity and adopting healthy lifestyles might help prevent the disease [3]. Most patients who presented at an early stage with the symptom of abnormal vaginal bleeding [3] are given a fair prognosis; however, a subset of patients suffer from poor outcomes despite aggressive treatment. To help guide treatment, the traditional Bokhman’s dualistic model [7] stratifies EC patients into type I and type II based on clinical and histopathological factors (such as cell types and tumor grading). Type I EC is typically low-grade and hormone-sensitive, and has a favorable prognosis [3,4]. In contrast, type II EC is high-grade, typically negative in hormone receptors, and has a poorer prognosis [7]. In a recent European population-based study [8], the age distributions among the two types of EC were similar.

Nevertheless, there is often a low interobserver agreement among pathologists regarding high-grade EC [9,10]. The molecular classification stated by The Cancer Genome Atlas (TCGA) in 2013 improved risk stratification independent of histopathology with better reproducibility [11,12,13]. Apart from the extremely good and poor outcomes of the molecular subtypes of POLE and p53 aberration [14], respectively, a majority of patients were in the group with no specific molecular profile or microsatellite instability (MSI) with intermediate risk [15]. It appears that the biological diversities seen in EC are not fully expressed using existing classification methods, therefore, additional markers to aid in risk stratification may be of clinical relevance.

Tumor-infiltrating lymphocytes (TILs), representing the host immunity against cancer cells, have emerged as a prognostic marker in various malignancies, such as melanoma, breast cancer, and EC [16,17]. TILs have been associated with high-grade histology, as well as molecular subtypes of mismatch repair (MMR) deficiency, which reflect an increase in immunogenicity induced by neoantigens in response to high mutational load [18]. However, whether TILs are associated with patient outcomes independent of histology factors and MMR status remains unclear [19,20]. In this study, we aimed to investigate the association between TILs and survival outcomes in patients with primary EC.

## 2. Materials and Methods

**Figure 1:**
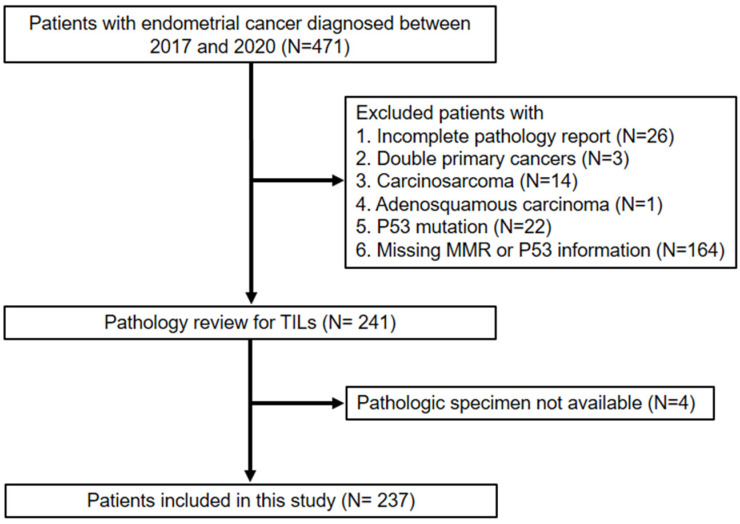
Selection of study population. MMR, mismatch repair. TILs, tumor-infiltrating lymphocytes.

This retrospective cohort study was approved by the Institutional Review Board of Taichung Veterans General Hospital (approval number: CE21444B). Figure 1 shows the selection of the study population. Among the 471 patients with primary EC consecutively diagnosed between 2017 and 2020, 230 were excluded due to incomplete pathology reports (*n* = 26), double primary cancers (*n* = 3), carcinosarcoma (*n* = 14), adenosquamous carcinoma (*n* = 1), p53 mutation (*n* = 22), and missing MMR or p53 information (*n* = 164). An independent pathologist reviewed the pathology reports to determine TILs. Ultimately, a total of 237 patients were included in our analyses.

All patients in this study underwent a standard staging operation involving total hysterectomy and bilateral salpingo-oophorectomy, with or without pelvic/para-aortic lymph node dissection at the discretion of the treating surgeon. Adjuvant chemotherapy and radiotherapy according to current evidence and guidelines were applied to patients with a high-risk profile after discussion in a tumor board consensus meeting. Relevant information surrounding pathological parameters (including cell type, tumor size, myometrial infiltration, cervical invasion, lymphovascular space invasion, lymph node and other metastasis, pathology stage, and status of estrogen receptor, progesterone receptor, MMR, and p53 mutation) was obtained from the electronic medical records.

**Figure 2:**
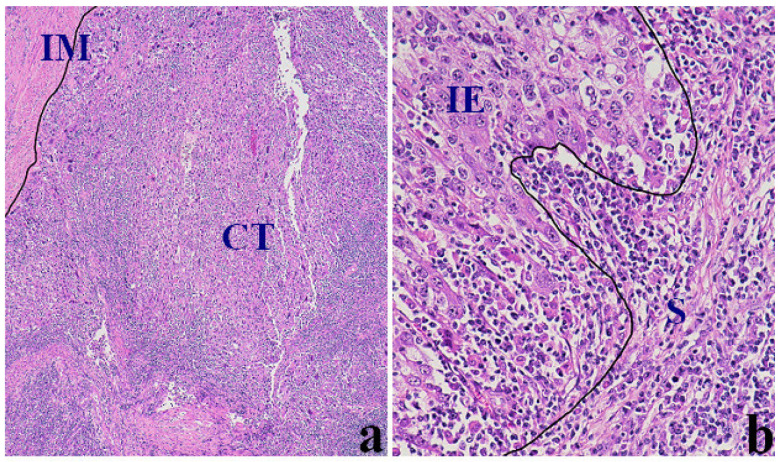
TILs in endometrial cancer: Typical hematoxylin and eosin sections of EC in ×40 (**a**) and ×200 (**b**) magnification, showing intense infiltration of the tumor epithelia and stroma by TILs. CT, central tumor. IE, intraepithelial. IM, invasive margin. S, stroma. TILs, tumor-infiltrating lymphocytes.

For the quantification of TILs, an independent pathologist who was blinded to the study patients’ pathologic stage and clinical outcome reviewed the pathologic slides. We followed the method introduced by the International Immuno-Oncology Biomarkers Working Group in 2017 for the standardization of TILs interpretation with hematoxylin and eosin (H&E) stain in solid tumors [21]. The H&E-stained slides were evaluated for TILs within the borders of the tumor, both in the central tumor (CT) and at the invasive margin (IM). The invasive margin was defined as the connective tissue just beneath the epithelial layer within 1 mm (Figure 2a). The localization of TILs was determined as intraepithelial (IE) or stroma (S) [21] (Figure 2b). The cut-off value to determine TIL (+) was 40 and 100 lymphocytes per 10 high-power fields, respectively, for intraepithelial and stroma TILs [22].

The outcomes of interest included both progression-free survival (PFS) and overall survival (OS). The status of disease progression and survival was confirmed by 30 April 2021. An abdominal CT scan was usually conducted every 3–6 months to assess the disease status of each patient. The progression of the disease was confirmed by the treating physician, with this information being retrieved from each patient’s electronic medical records. PFS was defined as the period from diagnosis of the disease to the date of documented disease progression, death, or last follow-up. OS was defined as the period from diagnosis of the disease to the date of death or last follow-up.

### Statistical Analysis

Between-group differences in baseline characteristics were compared using the independent *t*-test and chi-square test for continuous and categorical variables, respectively. The Kaplan–Meier method was used to determine curves of PFS and OS according to the TILs of the study population, and in relevant subgroups (low-grade vs. high-grade, MMR-proficient vs. MMR-deficient). Cox proportional hazard analysis was conducted to determine the effect of TILs on disease progression or death and all-cause mortality, with adjustments made for age, body mass index, nulliparous, pathology stage, lymphovascular space invasion, estrogen receptor, and progesterone receptor-positives, radiotherapy, and chemotherapy. We conducted all statistical analyses using the Statistical Package for Social Sciences (IBM SPSS version 22.0; International Business Machines Corp., Armonk, NY, USA). A *p*-value of less than 0.05 was considered statistically significant.

## 3. Results

**Figure 3:**
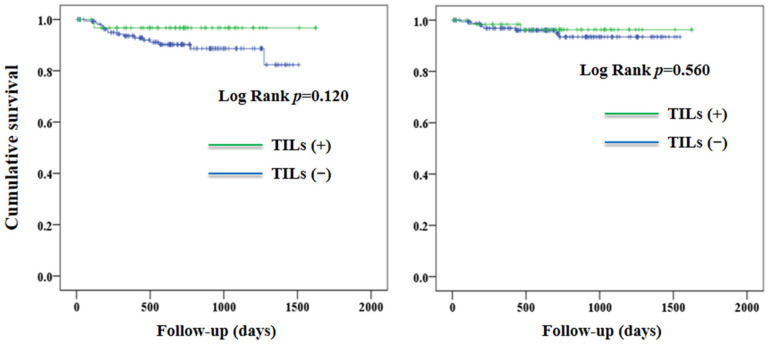
Progression-free survival (**left panel**) and overall survival (**right panel**) according to TILs. TILs, tumor-infiltrating T-lymphocytes.

Table 1 shows the clinical and pathological characteristics of the study population according to TILs [TILs (+) vs. TILs (−)]. There were no significant between-group differences in the baseline characteristics, including the status of MMR. After a median follow-up duration of 1.82 years, 18 patients experienced disease progression or death (10 patients died). Overall, patients with TILs (+) were not associated with a significantly better PFS (*p* = 0.120) or OS (*p* = 0.560) when compared to those with TILs (−) (Figure 3).

**Figure 4:**
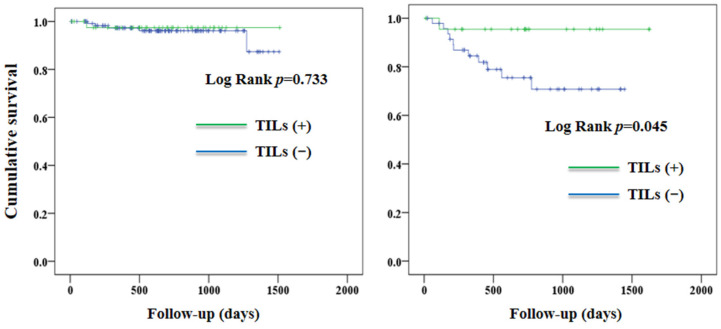
Progression-free survival in patients with low-grade (**left panel**) and high-grade (**right panel**) endometrial cancer according to TILs. TILs, tumor-infiltrating lymphocytes.

**Figure 5:**
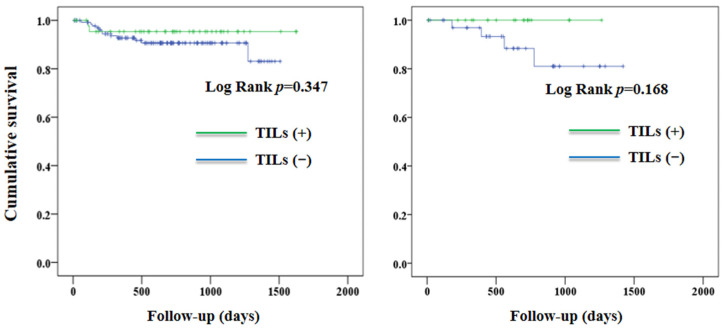
Progression-free survival in patients with MMR-proficient (**left panel**) and MMR-deficient (**right panel**) endometrial cancer according to TILs. MMR, mismatch repair. TILs, tumor-infiltrating T-lymphocytes.

Figure 4 shows the curves of PFS according to the TILs in patients with low-grade and high-grade EC. In patients with low-grade EC, there was no significant between-group difference in PFS (*p* = 0.733). In contrast, TILs (+) were associated with a better PFS (*p* = 0.045) in patients with high-grade EC. The effect of TILs on PFS was not observed in patients who were either MMR-proficient (*p* = 0.347) or MMR-deficient (*p* = 0.168) (Figure 5).

Table 2 shows the effect of TILs [TILs (+) vs. TILs (−)] on disease progression or death and all-cause mortality. Patients with TILs had a non-significantly lower risk of disease progression or death after adjustment for confounding factors (hazard ratio 0.358, 95% CI 0.076 to 1.684, *p* = 0.193, Table 2). This finding was consistent in patients with high-grade disease and those that were MMR-deficient. There was no significant effect of TILs on all-cause mortality (hazard ratio 0.907, 95% CI 0.128 to 6.423, *p* = 0.922).

## 4. Discussion

In this retrospective cohort study, we observed that TILs were not associated with any significant improvement in PFS and OS in patients with EC (Figure 2). Nevertheless, the association between TILs and PFS was not consistent in patients having different histology grades. TILs were associated with a better PFS in patients with high-grade EC, but not in those with low-grade tumors (Figure 3). Our findings suggest that the effect of TILs on PFS in patients with EC may be affected by histology grades.

TILs have been considered as being a marker of the host immune response against cancer cells [16]. Similar to findings in patients with ovarian cancer [23,24], TILs were associated with improved outcomes in patients with EC [25,26,27,28], despite inconsistent results in limited studies [17]. It is interesting to note that the presence of TILs was more frequently observed in patients with high-grade EC [18]. We found that TILs were associated with PFS in patients with high-grade EC. This finding was in line with the previous literature regarding patients with ovarian [29] and endometrial [30] cancer. In a study [31] of 90 patients diagnosed with EC, TILs were associated with a better survival rate for those with high-grade disease. These results suggest that there is an association between TILs and disease outcomes in patients with high-grade malignancies, which is an issue that merits further investigation and studies.

The difference in the association between TILs and PFS in patients with high-grade and low-grade EC could potentially be explained by the different types of TILs. Willvonseder B et al. [18] recently reported that CD3+ and CD8+ T cells were frequently observed in high-grade EC. In contrast, regulatory T cells were predominantly observed in low-grade tumors. In a previous review [24], CD3+ and CD8+ TILs seen in solid tumors had been associated with improved survival, whereas regulatory TILs were not linked to any survival benefits. These findings may help to explain our results that TILs were associated with better PFS in patients with high-grade EC, but not in those with low-grade tumors (Figure 3). Unfortunately, we did not differentiate the cell types of TILs in this study. This issue deserves further investigation in future studies.

The association between TILs and patient survival was also examined in patients having a different status of MMR. We excluded patients with a p53 aberration from our analyses. Given the small proportion of POLE (~10%) in patients with EC [14], the subgroups of MMR-proficient and MMR-deficient likely represented the copy number low and MSI population, respectively, according to the molecular classification of TCGA [14]. A higher number of CD3+ and CD8+ TILs was noted in tumors with MSI than in those with microsatellite stability (MSS) [32,33]. This finding was explained by the immune recognition of tumor-specific neoantigens in hypermutated tumors [34,35]. Nevertheless, it is not yet clear whether the association between TILs and outcomes in patients with MSS differed from that in patients with MSI. We did not observe an association between TILs and patient outcomes in the MMR-proficient and MMR-deficient subgroups (Figure 4). There was no association between TILs and survival when patients were grouped according to TCGA molecular classification [20]. These findings suggest that TILs may not be an important factor when accounting for the differences in outcomes between TCGA molecular subtypes.

Our study has several limitations. First, this was a retrospective cohort study with a relatively short follow-up duration (1.82 years), and few adverse outcomes (8 patients experienced disease progression and 10 patients died). Second, we did not confirm patients with POLE, as POLE sequencing was not routinely performed. Third, we did not differentiate the cell types of TILs (such as CD3+, CD8+, and regulatory T cells), or the density of these cells in the normal tissue adjacent to the tumor. These factors may have affected patient outcomes. The null associations between TILs and patient outcomes in the Cox proportional hazard analyses (Table 2) may be partly attributed to the aforementioned limitations. Given the paucity of data available on the association between TILs and survival outcomes in patients with EC, our findings are clinically relevant despite these limitations.

In summary, TILs were found to be associated with a better PFS in patients with high-grade EC, but not in those with low-grade tumors. No associations between TILs and outcomes were observed in patients who were MMR-proficient or MMR-deficient. While TILs may be a potential prognostic marker in patients with high-grade EC, our findings still merit further investigation.

## Figures and Tables

**Table 1:** Characteristics of the study population.

Variables	TILs (−)	TILs (+)	*p*
Number of patients	172	65	
Age, years	54.4 ± 10.5	55.7 ± 9.5	0.391
Body mass index, kg/m^2^	26.6 ± 5.9	25.6 ± 5.9	0.257
Nulliparous, *n* (%)	50 (29.1)	21 (32.3)	0.627
Cell type, *n* (%)			0.353
Endometrioid	168 (97.7)	62 (95.4)	
Others	4 (2.3)	3 (4.6)	
High-grade, *n* (%) ^a^	49 (28.5)	23 (35.4)	0.303
Tumor size ≥ 20 mm, *n* (%)	133 (77.3)	56 (86.2)	0.131
Myometrial infiltration ≥ 50%, *n* (%)	51 (29.7)	17 (26.2)	0.595
Lymphovascular space invasion, *n* (%)	50 (29.1)	21 (32.3)	0.652
Lymph node metastasis, *n* (%)	18 (10.5)	10 (15.4)	0.218
Pathology stage, *n* (%)			0.795
Stage 1	135 (78.5)	50 (76.9)	
Stage 2–4	37 (21.5)	15 (23.1)	
Estrogen receptor-positive, *n* (%)	164 (95.3)	59 (90.8)	0.168
Progesterone receptor-positive, *n* (%)	151 (87.8)	55 (84.6)	0.123
MMR-deficient, *n* (%)	36 (20.9)	19 (29.2)	0.177
Radiotherapy, *n* (%)	41 (23.8)	17 (26.2)	0.711
Chemotherapy, *n* (%)	33 (19.2)	16 (24.6)	0.357

Values are mean ± SD or *n* (%). ^a^ Grade 3 endometroid or non-endometrioid cell type. MMR, mismatch repair. TILs, tumor-infiltrating T-lymphocytes.

**Table 2:** Effect of TILs on disease progression or death and all-cause mortality.

Independent variable: TILs (+ vs. −)	Hazard Ratio (95% CI) ^a^	*p*
Overall		
Disease progression or death	0.358 (0.076, 1.684)	0.193
All-cause mortality	0.907 (0.128, 6.423)	0.922
In high-grade subgroup		
Disease progression or death	0.265 (0.027, 2.553)	0.250
All-cause mortality	0.682 (0.051, 9.106)	0.772
In MMR-deficient subgroup		
Disease progression or death	0.012 (0, >999)	0.775
All-cause mortality	NA	NA

^a^ Adjusted for age, body mass index, nulliparous, pathology stage, lymphovascular space invasion, estrogen receptor and progesterone receptor-positive, radiotherapy, and chemotherapy. MMR, mismatch repair; TILs, tumor-infiltrating T-lymphocytes; NA, Not applicable.

## Data Availability

The data presented in this study are available on request from the corresponding author. The data are not publicly available due to privacy/ethical restrictions.

## References

[1] Sung H., Ferlay J., Siegel R.L., Laversanne M., Soerjomataram I., Jemal A., Bray F. (2021). Global Cancer Statistics 2020: GLOBOCAN Estimates of Incidence and Mortality Worldwide for 36 Cancers in 185 Countries. CA Cancer J. Clin..

[2] Gu B., Shang X., Yan M., Li X., Wang W., Wang Q., Zhang C. (2021). Variations in incidence and mortality rates of endometrial cancer at the global, regional, and national levels, 1990–2019. Gynecol. Oncol..

[3] Amant F., Moerman P., Neven P., Timmerman D., Van Limbergen E., Vergote I. (2005). Endometrial cancer. Lancet.

[4] Takenaka K., Olzomer E.M., Hoehn K.L., Curry-Hyde A., Jun Chen B., Farrell R., Byrne F.L., Janitz M. (2022). Investigation of circular RNA transcriptome in obesity-related endometrial cancer. Gene.

[5] Blüher M. (2019). Obesity: Global epidemiology and pathogenesis. Nat. Rev. Endocrinol..

[6] Chatterji S., Byles J., Cutler D., Seeman T., Verdes E. (2015). Health, functioning, and disability in older adults--present status and future implications. Lancet.

[7] Bokhman J.V. (1983). Two pathogenetic types of endometrial carcinoma. Gynecol. Oncol..

[8] Rodríguez-Palacios D.Á., Colorado-Yohar S.M., Velten M., Vaamonde-Martín R.J., Ballesta M., Chirlaque M.D. (2022). Incidence and Trend of Type I and II Endometrial Cancer in Women from Two Population-Based European Cancer Registries (1998–2012). Int. J. Environ. Res. Public Health.

[9] Gilks C.B., Oliva E., Soslow R.A. (2013). Poor interobserver reproducibility in the diagnosis of high-grade endometrial carcinoma. Am. J. Surg. Pathol..

[10] Han G., Sidhu D., Duggan M.A., Arseneau J., Cesari M., Clement P.B., Ewanowich C.A., Kalloger S.E., Köbel M. (2013). Reproducibility of histological cell type in high-grade endometrial carcinoma. Mod. Pathol..

[11] Levine D.A., The Cancer Genome Atlas Research Network (2013). Integrated genomic characterization of endometrial carcinoma. Nature.

[12] Talhouk A., McConechy M.K., Leung S., Li-Chang H.H., Kwon J.S., Melnyk N., Yang W., Senz J., Boyd N., Karnezis A.N. (2015). A clinically applicable molecular-based classification for endometrial cancers. Br. J. Cancer.

[13] Stelloo E., Nout R.A., Osse E.M., Jürgenliemk-Schulz I.J., Jobsen J.J., Lutgens L.C., van der Steen-Banasik E.M., Nijman H.W., Putter H., Bosse T. (2016). Improved Risk Assessment by Integrating Molecular and Clinicopathological Factors in Early-stage Endometrial Cancer-Combined Analysis of the PORTEC Cohorts. Clin. Cancer Res..

[14] Urick M.E., Bell D.W. (2019). Clinical actionability of molecular targets in endometrial cancer. Nat. Rev. Cancer.

[15] Talhouk A., McAlpine J.N. (2016). New classification of endometrial cancers: The development and potential applications of genomic-based classification in research and clinical care. Gynecol. Oncol. Res. Pract..

[16] Zal T., Chodaczek G. (2010). Intravital imaging of anti-tumor immune response and the tumor microenvironment. Semin. Immunopathol..

[17] Guo F., Dong Y., Tan Q., Kong J., Yu B. (2020). Tissue Infiltrating Immune Cells as Prognostic Biomarkers in Endometrial Cancer: A Meta-Analysis. Dis. Markers.

[18] Willvonseder B., Stögbauer F., Steiger K., Jesinghaus M., Kuhn P.H., Brambs C., Engel J., Bronger H., Schmidt G.P., Haller B. (2021). The immunologic tumor microenvironment in endometrioid endometrial cancer in the morphomolecular context: Mutual correlations and prognostic impact depending on molecular alterations. Cancer Immunol. Immunother..

[19] Miyamoto M., Hada T., Ishibashi H., Iwahashi H., Kakimoto S., Suzuki R., Sakamoto T., Matsuura H., Tsuda H., Takano M. (2021). A New Model to Improve the Prediction of Prognosis of Endometrial Carcinoma by Combining Traditional Classification with the Presence of Tumor-infiltrating Lymphocytes. Anticancer Res..

[20] Talhouk A., Derocher H., Schmidt P., Leung S., Milne K., Gilks C.B., Anglesio M.S., Nelson B.H., McAlpine J.N. (2019). Molecular Subtype Not Immune Response Drives Outcomes in Endometrial Carcinoma. Clin. Cancer Res..

[21] Hendry S., Salgado R., Gevaert T., Russell P.A., John T., Thapa B., Christie M., Van De Vijver K., Estrada M.V., Gonzalez-Ericsson P.I. (2017). Assessing Tumor-Infiltrating Lymphocytes in Solid Tumors: A Practical Review for Pathologists and Proposal for a Standardized Method from the International Immuno-Oncology Biomarkers Working Group: Part 2: TILs in Melanoma, Gastrointestinal Tract Carcinomas, Non-Small Cell Lung Carcinoma and Mesothelioma, Endometrial and Ovarian Carcinomas, Squamous Cell Carcinoma of the Head and Neck, Genitourinary Carcinomas, and Primary Brain Tumors. Adv. Anat. Pathol..

[22] Shia J., Black D., Hummer A.J., Boyd J., Soslow R.A. (2008). Routinely assessed morphological features correlate with microsatellite instability status in endometrial cancer. Hum. Pathol..

[23] Leffers N., Gooden M.J., de Jong R.A., Hoogeboom B.N., ten Hoor K.A., Hollema H., Boezen H.M., van der Zee A.G., Daemen T., Nijman H.W. (2009). Prognostic significance of tumor-infiltrating T-lymphocytes in primary and metastatic lesions of advanced stage ovarian cancer. Cancer Immunol. Immunother..

[24] Zhang L., Conejo-Garcia J.R., Katsaros D., Gimotty P.A., Massobrio M., Regnani G., Makrigiannakis A., Gray H., Schlienger K., Liebman M.N. (2003). Intratumoral T cells, recurrence, and survival in epithelial ovarian cancer. N. Engl. J. Med..

[25] de Jong R.A., Leffers N., Boezen H.M., ten Hoor K.A., van der Zee A.G., Hollema H., Nijman H.W. (2009). Presence of tumor-infiltrating lymphocytes is an independent prognostic factor in type I and II endometrial cancer. Gynecol. Oncol..

[26] Čermáková P., Melichar B., Tomšová M., Zoul Z., Kalábová H., Spaček J., Doležel M. (2014). Prognostic significance of CD3+ tumor-infiltrating lymphocytes in patients with endometrial carcinoma. Anticancer Res..

[27] Kono-Sato T., Miyai K., Yamagishi Y., Miyamoto M., Takano M., Matsukuma S., Sato K., Tsuda H. (2022). Intraepithelial lymphocytes are indicators of better prognosis in surgically resected endometrioid-type endometrial carcinomas at early and advanced stages. BMC Cancer.

[28] Bounous V.E., Ferrero A., Campisi P., Fuso L., Pezua Sanjinez J.O.S., Manassero S., De Rosa G., Biglia N. (2022). Immunohistochemical Markers and TILs Evaluation for Endometrial Carcinoma. J. Clin. Med..

[29] Paijens S.T., Vledder A., Loiero D., Duiker E.W., Bart J., Hendriks A.M., Jalving M., Workel H.H., Hollema H., Werner N. (2021). Prognostic image-based quantification of CD8CD103 T cell subsets in high-grade serous ovarian cancer patients. Oncoimmunology.

[30] Kondratiev S., Sabo E., Yakirevich E., Lavie O., Resnick M.B. (2004). Intratumoral CD8+ T lymphocytes as a prognostic factor of survival in endometrial carcinoma. Clin. Cancer Res..

[31] Gooden M.J., de Bock G.H., Leffers N., Daemen T., Nijman H.W. (2011). The prognostic influence of tumour-infiltrating lymphocytes in cancer: A systematic review with meta-analysis. Br. J. Cancer.

[32] Howitt B.E., Shukla S.A., Sholl L.M., Ritterhouse L.L., Watkins J.C., Rodig S., Stover E., Strickland K.C., D'Andrea A.D., Wu C.J. (2015). Association of Polymerase e-Mutated and Microsatellite-Instable Endometrial Cancers with Neoantigen Load, Number of Tumor-Infiltrating Lymphocytes, and Expression of PD-1 and PD-L1. JAMA Oncol..

[33] Hussein Y.R., Weigelt B., Levine D.A., Schoolmeester J.K., Dao L.N., Balzer B.L., Liles G., Karlan B., Köbel M., Lee C.H. (2015). Clinicopathological analysis of endometrial carcinomas harboring somatic POLE exonuclease domain mutations. Mod. Pathol..

[34] Ino K., Yamamoto E., Shibata K., Kajiyama H., Yoshida N., Terauchi M., Nawa A., Nagasaka T., Takikawa O., Kikkawa F. (2008). Inverse correlation between tumoral indoleamine 2,3-dioxygenase expression and tumor-infiltrating lymphocytes in endometrial cancer: Its association with disease progression and survival. Clin. Cancer Res..

[35] Whiteside T.L. (2006). Immune suppression in cancer: Effects on immune cells, mechanisms and future therapeutic intervention. Semin. Cancer Biol..

